# Soluble Receptor Isoform of IFN-Beta (sIFNAR2) in Multiple Sclerosis Patients and Their Association With the Clinical Response to IFN-Beta Treatment

**DOI:** 10.3389/fimmu.2021.778204

**Published:** 2021-12-16

**Authors:** Pablo Aliaga-Gaspar, Isaac Hurtado-Guerrero, Nicolas Lundahl Ciano-Petersen, Patricia Urbaneja, Isabel Brichette-Mieg, Virginia Reyes, Jose Luis Rodriguez-Bada, Roberto Alvarez-Lafuente, Rafael Arroyo, Ester Quintana, Lluis Ramió-Torrentà, Ana Alonso, Laura Leyva, Oscar Fernández, Begoña Oliver-Martos

**Affiliations:** ^1^ Neuroimmunology and Neuroinflammation Group, Instituto de Investigación Biomédica de Málaga (IBIMA), Unidad de Gestión Clínica (UGC) Neurociencias, Hospital Regional Universitario de Málaga, Málaga, Spain; ^2^ Facultad de Medicina, Universidad de Málaga, Málaga, Spain; ^3^ Neuroinflammation Unit, Biotech Research and Innovation Centre (BRIC), Faculty of Health and Medical Sciences, Copenhagen Biocentre, University of Copenhagen, Copenhagen, Denmark; ^4^ Red Andaluza de Investigación Clínica y Traslacional en Neurología (Neuro-Reca), Málaga, Spain; ^5^ Grupo de Investigación de Factores Ambientales en Enfermedades Degenerativas, Instituto de Investigación Sanitaria del Hospital Clínico San Carlos (IdISSC), Madrid, Spain; ^6^ Red Española de Esclerosis Múltiple (REEM), Madrid, Spain; ^7^ Servicio de Neurología, Hospital Universitario Quirónsalud, Madrid, Spain; ^8^ Servicio de Neurología, Hospital Universitari de Girona Doctor Josep Trueta, Girona, Spain; ^9^ Girona Biomedical Research Institute (IDIBGI), Girona, Spain; ^10^ Medical Sciences Department, University of Girona, Girona, Spain; ^11^ Departmento de Farmacología, Facultad de Medicina, Universidad de Málaga, Málaga, Spain; ^12^ Departamento de Biología Celular, Genética y Fisiología, Área de Fisiología, Facultad de Ciencias, Universidad de Málaga, Málaga, Spain

**Keywords:** alternative splicing, soluble receptors, IFNAR, interferon beta, multiple sclerosis

## Abstract

**Purpose:**

Interferon beta receptor 2 subunit (IFNAR2) can be produced as a transmembrane protein, but also as a soluble form (sIFNAR2) generated by alternative splicing or proteolytic cleavage, which has both agonist and antagonist activities for IFN-β. However, its role regarding the clinical response to IFN-β for relapsing-remitting multiple sclerosis (RRMS) is unknown. We aim to evaluate the *in vitro* short-term effects and after 6 and 12 months of IFN-β therapy on sIFNAR2 production and their association with the clinical response in MS patients.

**Methods:**

Ninety-four RRMS patients were included and evaluated at baseline, 6 and 12 months from treatment onset. A subset of 41 patients were classified as responders and non-responders to IFN-β therapy. sIFNAR2 serum levels were measured by ELISA. mRNA expression for IFNAR1, IFNAR2 splice variants, MxA and proteases were assessed by RT-PCR. The short-term effect was evaluated in PBMC from RRMS patients after IFN-β stimulation *in vitro*.

**Results:**

Protein and mRNA levels of sIFNAR2 increased after IFN-β treatment. According to the clinical response, only non-responders increased sIFNAR2 significantly at both protein and mRNA levels. sIFNAR2 gene expression correlated with the transmembrane isoform expression and was 2.3-fold higher. While MxA gene expression increased significantly after treatment, IFNAR1 and IFNAR2 only slightly increased. After short-term IFN-β *in vitro* induction of PBMC, 6/7 patients increased the sIFNAR2 expression.

**Conclusions:**

IFN-β administration induces the production of sIFNAR2 in RRMS and higher levels might be associated to the reduction of therapeutic response. Thus, levels of sIFNAR2 could be monitored to optimize an effective response to IFN-β therapy.

## Introduction

Multiple sclerosis (MS) is the most prevalent chronic inflammatory autoimmune disease of the central nervous system, with a complex pathophysiology characterized by inflammation, demyelination, and axonal degeneration. Although in the last two decades the approval of several disease-modifying therapies have revolutionized the management of MS (1), IFN-β is still used as a first line therapeutic option because of benefit/risk profile and cost. A wide range of immunomodulatory effects have been associated to IFN-β, being able to reduce the antigen presentation, inhibit the proliferation of T cells, and shift cytokine production (2). However, despite the fact that this drug has been used for more than 25 years, its mechanism of action is not completely understood. It has been estimated that 20 to 50% of patients are non-responsive to the treatment (3), highlighting the need for biomarkers to predict treatment response.

Cytokine receptors are usually expressed by cells as transmembrane proteins, however, most of them are also secreted in soluble forms and can be detected in different human body fluids including the blood, the tears, the cerebrospinal fluid or the urine (4, 5). The generation of soluble receptors is an important mechanism by which the biological activity of cytokines is modulated due to their ability to bind them, acting as antagonists or agonists (6, 7), so that they can be used for therapeutic purposes (4).

There are two main mechanisms to generate soluble receptors: alternative splicing at the gene level and proteolytic cleavage at the protein level (8). On the one hand, alternative splicing is a mechanism to regulate gene expression that allows a single gene to code for multiple proteins isoforms and occurs in up to 94% of human genes (9). For instance, some isoforms of cytokine receptors generated by alternative splicing may lose the extracellular domain, which is subsequently secreted to the bloodstream (10). On the other hand, proteolytic cleavage of receptors on the cell surface is accomplished by a family of specialized enzymes called proteases, which result in the excision of the extracellular domain and its release into the bloodstream (11). Probably, the most important proteases in immunity and inflammation are metalloproteases of the ADAM family, notably ADAM17 (12, 13).

IFN-β is a cytokine that exerts its biological activity thought interaction with its cell surface receptor (IFNAR), which is composed of two functional subunits: IFNAR1 and IFNAR2, and the subsequent activation of the JAK-STAT signaling pathway (14). The IFNAR2 subunit has a soluble isoform (sIFNAR2) that can be generated by alternative splicing of the human IFNAR2 gene giving a transcript that lacks the transmembrane and cytoplasmic domain that was cloned in human and mouse (15, 16). Besides, the IFNAR2 subunit can be cleaved by specific proteases such as TNF-alpha converting enzyme (known as TACE or ADAMS) that releases the extracellular domain (17), as well as by presenilins (PSEN) that release the intracellular domain (18). As a result of both mechanisms, sIFNAR2 can be detected in body fluids (19) and has been found to be expressed at levels 10-fold higher than the transmembrane IFNAR2 transcripts in most mouse tissues (20).

Although type I interferon responses and their regulation are widely described in autoimmune diseases, including MS, the presence and action of the sIFNAR2 has not been explored, despite its ability of binding endogenous and exogenous IFN-β and modulating their activity (20–22). This capacity of modulate the IFN-β activity and its accessibility in serum makes sIFNAR2 an attractive target molecule to be considered in MS, due to the key role of endogenous IFN-β in the pathophysiology of MS and even more if we consider the use of exogenous IFN-β as a treatment. Accordingly, we previously demonstrated that untreated-MS patients had lower sIFNAR2 levels than healthy controls, patients with other inflammatory neurological diseases and MS patients treated with IFN-β, highlighting its value as a diagnostic biomarker for MS. Moreover, we proved that patients treated with IFN-β have higher circulating levels of sIFNAR2, in contrast to those treated with Glatiramer Acetate (GA) or Natalizumab (23).

Our aim was to assess the *in vitro* short-term effects and after 6 and 12 months of IFN-β treatment on the circulating sIFNAR2 levels and on the gene expression of the different IFNAR2 splice variants in MS patients, as well as their relation with the therapeutic response to IFN-β.

## Material and Methods

### Participant Centers and Subjects

Fifty-nine RRMS patients, defined according to the revised McDonald criteria (24) were recruited from the MS Unit at Malaga Regional Hospital (Malaga, Spain), before to start IFN-β treatment, and were followed at 6 and 12 months after IFN-β treatment onset (Cohort 1). Forty-one of them whose data of relapses, EDSS progression and MRI activity were available, were classified after one year of treatment as responders (R) or non-responders (NR) according to the Rio Score (25). Nineteen patients (46.35%) were considered non-responders according to the occurrence of two or three positive variables during the first year of therapy (at least one relapse during the first year of therapy; an increase of at least one point in the EDSS score during the first year of therapy (confirmed at 6 months); three or more active lesions (either new or enlarging T2 lesions compared to baseline MRI scan or gadolinium-enhancing lesions) on the MRI performed after 1 year of therapy); the remaining twenty-two (53.65%) were considered responders.

An independent cohort including 25 MS patients from Dr. Josep Trueta University Hospital, Girona (Spain) and 10 MS patients from Hospital San Carlos, Madrid (Spain) was also analyzed at baseline and at 6 and 12 months after treatment onset for validation of the results (Cohort 2).

All participants in the study gave informed consent and the protocols were approved by the institutional ethical committee (Comité de Ética de la Investigación Málaga Nordeste. Project identification: DTS1800045).

**Table 1**

**Table 1:** Clinical and demographic data.

	Cohort 1	Cohort 2	Responders^†^	Non responders^†^	P-value*
*N*	59	35	22	19	–
Female/Male	38/21	25/10	11/11	12/7	n.s.
EDSS score at baseline	1,08 ± 1,33	1,70 ± 0,87	0,97 ± 1,46	1,13 ± 1,21	0,039
IFN-β type (a)	12/7/32/2/0	4/22/8/0/3	4/0/18/0/0	4/4/10/1/0	–
EDSS score after one year of therapy	–	–	0,93 ± 1,48	1,71 ± 1,21	0,008
MRI activity (b)	–	–	13/1	8/3	<0,001
Number of relapses after one year of therapy	–	–	0 ± 0	1,26 ± 1,09	<0,001

(a) Treatment type: Avonex/Betaferon/Rebif/Extavia/Plegridy.

(b) MRI activity: Negative/Positive.

*P-values obtained by chi-square test (gender, MRI activity) or Mann-Whitney test.

^†^From cohort 1.

n.s. (Non significant).

### Sample Collection

For serum determinations, 3 ml of peripheral blood were collected in serum-separating tubes basally and after 6 and 12 months of treatment onset. For gene expression studies, peripheral blood samples were collected in EDTA tubes, only in patients from cohort 1. In all cases, the time elapsed between the administration of IFN-β and sampling was of at least 24 hours.

Samples were processed following standard procedures and frozen immediately after reception by the Malaga Hospital-IBIMA Biobank, as part of Andalusian Public Health System Biobank. PBMC were isolated using a ficoll-hypaque gradient, as described in the supplier’s protocol (ICN Biomedicals Inc.). After that, cells were cryopreserved in RPMI-1640- 10% DMSO until use for RNA extraction.

### Quantification of Soluble IFNAR2 by ELISA

sIFNAR2 levels were quantified with an ELISA previously developed and validated in our laboratory (26). First, a recombinant sIFNAR2 protein was cloned, expressed and purified and then used as standard curve in the ELISA (23, 26). Briefly, the plates were coated with 0.2-μg rabbit polyclonal anti-human IFNAR2 antibody (Abnova) overnight. After washing, the wells were blocked with a casein blocking buffer [2 hours, room temperature (RT)]. Then, 50 μL of each point of the standard curve or serum samples (diluted in half) were added to the wells in duplicate, and incubated for 1 hour. Mouse polyclonal anti-human IFNAR2 antibody (1 μg/mL in assay buffer; Abnova) was added and incubated (1 hour, RT). After washing again, 50-μL horseradish peroxidase (HRP)-conjugated goat anti-mouse IgG (H+L) adsorbed against human immunoglobulins was added and incubated (1 hour, RT). After three additional washes, 100 μL/well 3,3′,5,5′-tetramethylbenzidine (TMB) One Component HRP Microwell Substrate was added and incubated (10–15 minutes in the dark). Color development was done by adding 50 μL/well 1 N H2SO4. Optical density (OD) was measured at 450 nm in a VersaMax™ ELISA Microplate Reader (Molecular Devices)

Sample analysis. Each assay included a standard curve, two quality controls, and a negative control. sIFNAR2 concentration was calculated by OD interpolation from the samples and controls in the standard curve, that was established using a four-parameter curve fitting model by the SoftMax^®^ Pro Software (Molecular Devices). Sample measurement was considered acceptable if the intra and inter assay coefficients of variation (CV) were <10% and <20% respectively (27). Here, the average of the intra and inter-assay CV was 3.6% and 13%, respectively.

### Isolation of RNA and Quantitative Reverse Transcriptase-PCR

Total RNA was isolated from PBMC fromMS patients following the instructions of Aurum Total RNA Mini KIT (BioRad). RNA concentration and purity were analyzed in a NanoDrop 2000 Spectrophotometer. Total RNA (500 ng) was reverse‐transcribed using the M-MLV retrotranscriptase (Sigma). Samples were run in the same reaction, and the resulting cDNA was controlled for purity and transcription efficiency on the NanoDrop.

RT² qPCR Primer Assay for Human IFNAR1, IFNAR2, MxA and PSEN 1 and 2 from Qiagen were used. Primers for ADAM17 and sIFNAR2 were designed with Primer3 (http://frodo.wi.mit.edu/primer3) and ordered to Isogen Life Science (**Supplementary Table 1**). For sIFNAR2 the design was carried out by taking into account that this isoform is the product of an alternative splicing, therefore, the primers must hybridize in a specific region, so that the resulting amplified product is exclusive to this isoform (**Supplementary Figure 1**). RT‐qPCR was performed in duplicate using iTaq Universal SYBR Green SMX (BioRad), and was run on a 7500 Fast Real‐Time PCR System (Applied Biosystems). Relative expressions of genes were analysed by comparison of ΔΔCt values using GAPDH as raeference gene. MIQE Guidelines were considered for real time experiments (28).

### Quantification of IFN-β in Serum by Luminex

IFN-β serum levels were assessed basally and after treatment using a customized Procartaplex Immunoassay (Thermo Fisher). 25 µL per well of antibody coupled beads and 50 µL per well of diluted patient serum were added into a 96 well plate and incubated overnight at 4°C. Each assay included a standard curve and all samples were performed in duplicate. Plates were processed following manufacturers’ instructions and then read with a Bio-Plex^®^ 200 system (Bio-Rad). The data obtained were processed with the Bioplex Manager 6.0 software (Bio-Rad) using a five-parameter curve fitting algorithm to analyse them.

### Neutralizing Antibodies (NABs) Against IFN Beta by Cytopathic Effect Test (CPE Test)

Sera of IFNβ-treated patients were tested for the presence of NAbs against the three commercial forms of IFN-β (IFN-β 1a (Avonex, Biogen, Inc.), IFN-β 1b (Betaferon, Bayer Pharma AG) or IFN-β 1a (Rebif, Merck Serono Ltd) by a calibrated antiviral CPE test, as previously described by 29. Briefly, A549 cells were seeded in 96 well plates in 100 μl of DMEM medium supplemented with 2% FCS. After 24 h, serial dilutions of the patient sera were incubated for 1 h with IFN-β, and without IFN-β to control the presence of endogenous IFN-β in the patient serum. These dilutions were added to the A549 cell culture, seeded the day before. Each plate included a viral control which did not contain IFN-β, a cell control which did not contain virus, and a standard of IFN-β. After 24 h incubation, cells were infected with Encephalomyocarditis virus (EMCV) (except for the cell control). Twenty-four hours later, the cells were stained with crystal violet and the absorbance was read at 630 lambdas in a spectrophotometer. The neutralization titre was calculated according to 30 and expressed in 10-fold reduction units per millilitre (TRU/mL). Titres ≥20 TRU/mL were considered as positive.

### Cell Cultures in the Presence of Recombinant IFN-β

PBMC isolated from 7 randomly untreated MS patients were thawed and suspended (1x10^6^ cells mL-1) in pre-warmed RPMI-1640 medium, supplemented with 2 mM l-glutamine (MP Biomedicals, Irvine, CA, USA), 10% Fetal Bovine Serum (FBS) and 0.032 mg mL-1 gentamicin (Normon). Cells were washed by centrifugation and resuspended in RPMI-1640 complete medium with 2% FBS.

To evaluate the ability of recombinant IFN-β to induce the production of sIFNAR2, the cells were stimulated with 20 units/mL of IFN-β (Avonex) during 8 and 24 hours. A non-stimulated control was included each time. After that, the cells were collected for RNA extraction and cDNA conversion, as explained above. sIFNAR2, IFNAR2 and MxA were measured by real time PCR. MxA was considered as positive control since it is well known that its expression is clearly induced after IFN-β stimulation (31).

### Statistical Analysis

As a non-normal distribution was established in the Kolmogorov–Smirnov test, non-parametric tests were used for comparison between groups. The results are expressed as median and interquartile ranges.

For each comparison, all groups were assigned equal sample sizes. The samples were not randomized, and untreated and treated samples were run in parallel in order to minimize the inter-assay variation. Wilcoxon Rank test (related samples analysis) was used to compare the variables (sIFNAR2, IFN-β and the gene expression of IFNAR1, IFNAR2 sIFNAR2, MxA and PSEN 1 and 2) before and after treatment.

To analyze the association or interdependence between the gene expression of IFN-β subunits and MxA, and between gene expression and protein levels, Spearman’s Rho correlation analysis was used. Statistical significance was set at p <0.05. Figures have been created with GraphPad Prism5 Software. *In vitro* results were represented using log10 for normalizing the data.

## Results

### Serum Levels of Soluble IFNAR2 Increase With Long IFN-β Treatment

**Figure 1:**
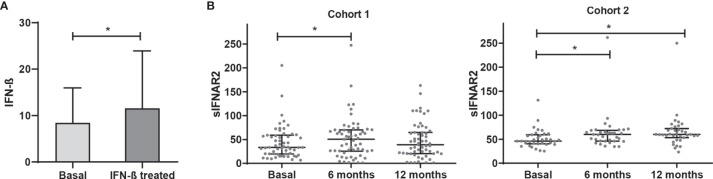
IFN-β levels and longitudinal assessment of serum sIFNAR2 levels in MS patients. **(A)** Serum IFN-β levels measured in MS patients basally and after IFN-β therapy onset (N=59). Significant p-value is shown with asterisk. **(B)** Graphs comparing serum sIFNAR2 levels at baseline and after 6 and 12 months of IFN-β therapy onset in two independent cohorts (Cohort 1 N=59, Cohort 2 N=35). Each point represents an individual, and horizontal bars indicate the median values and interquartile ranges. Significant p-values are shown with asterisk.

In the cohort 1, the first step was the confirmation that IFN-β treated patients increased circulating levels of IFN-β compared to the levels before the treatment onset (p=0.001) (**Figure 1**). Also, it was demonstrated that all the patients included were negative for NABs against the molecule of the IFN-β that were receiving.

Regarding sIFNAR2, we found increased levels at 6- and 12-months follow-up after IFN-β exposure compared to baseline levels, with significant differences at 6 months (p=0.008). This increase of sIFNAR2 levels was replicated in the cohort 2, being statistically significant at 6 months (p=0.0001) and at 12 months (p=0.0001).

Considering all the patients, serum sIFNAR2 levels were increased in 70.4% of the patients at 6 months of treatment and remained increased at 12 months in 60.3% of them. In **Supplementary Figure 2** the ΔsIFNAR2 has been represented for each patient.

**Figure 1**, **Table 2**

**Table 2:** Serum sIFNAR2 levels in MS patients.

Cohort 1/Cohort 2	Basal	6 Months	12 Months
*N*	59/35	59/35	59/35
25% Percentile	19,59/40,44	25,61/45,94	20,41/53,3
Median	33,43/46,01	50,6/60,04	38,97/59,97
75% Percentile	59,16/59,44	70,16/68,44	65/72,15

### Serum Levels of Soluble IFNAR2 According to the IFN-β Response

Patients from cohort 1 whose data was available, were classified as responders (N=22) and non-responders (N=19) according to the Rio Score (25). In the follow-up study, responders maintained stable serum sIFNAR2 levels, however, non-responder patients showed a significant increase after 6 months of treatment (p=0.018), returning to baseline levels at 12 months.

**Figure 2:**
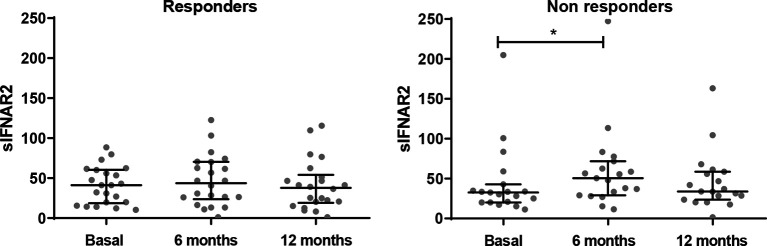
Longitudinal assessment of serum sIFNAR2 levels in responder and non-responder patients. Graphs comparing serum sIFNAR2 levels at baseline and after 6 and 12 months of IFN-β therapy onset in responder (N=22) and non-responder patients (N=19). Each point represents an individual, and horizontal bars indicate the median values and interquartile ranges. Significant p-values are shown with asterisk.

**Figure 2**, **Table 3**

**Table 3:** Serum sIFNAR2 levels in MS patients according to IFN beta response.

Responders/Non-responders	Basal	6 Months	12 Months
*N*	22/19	22/19	22/19
25% Percentile	16,52/20,19	18,8/28,99	16,5/23,64
Median	37,61/32,58	35,58/50,6	30,91/33,83
75% Percentile	61,2/42,71	70,38/71,7	59,62/58,72

### sIFNAR2 Gene Expression Increases With IFN- β Treatment

Forty-one MS patients from cohort 1, who had available PBMC samples at baseline and after 6 of months of treatment onset, were included for the gene expression study of IFNAR1, the transmembrane and soluble transcripts of IFNAR2, and MxA gene. Although there was an increase in IFNAR1 and IFNAR2 gene expression after IFN-β treatment, those changes did not reach statistical significance. However, the expression of sIFNAR2 transcript and MxA gene were significantly increased after IFN-β treatment (p=0.017 and p<0.001 respectively).

**Figure 3**

**Figure 3:**
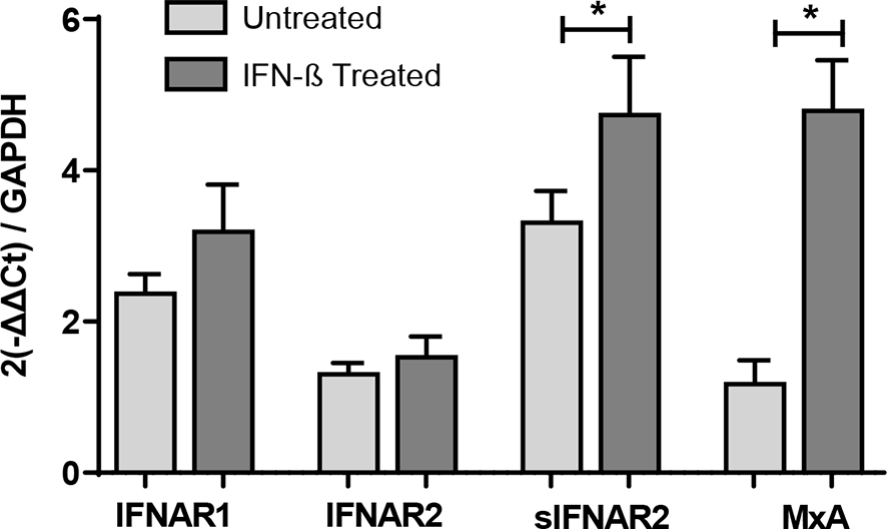
Longitudinal assessment of IFNAR1, IFNAR2, sIFNAR2 and MxA gene expression. RNA relative expression of IFNAR1, IFNAR2, sIFNAR2 and MxA in PBMC from MS patients before and after 6 months of IFN-β therapy onset (N=41), assessed by real time-PCR using GAPDH as raeference gene. Significant p-values are shown with asterisk.

### IFNAR2 Splice Variants Under IFN-β Treatment

Transmembrane IFNAR2 and sIFNAR2 are products of an alternative splicing of IFNAR2 gene. The primer design carried out allowed the quantification of both splice variants independently. A strong positive correlation between the gene expression levels of IFNAR2 and sIFNAR2 before (r=0.636 p< 0.001) and after the IFN-β treatment (r=0.749 p<0.001) was observed.

Interestingly, the expression of sIFNAR2 was significantly greater compared to the transmembrane isoform before (1.35 vs 3.17; p<0.001) and after IFN-β treatment (1.65 vs 4.98 p<0.001).

**Figure 4**

**Figure 4:**
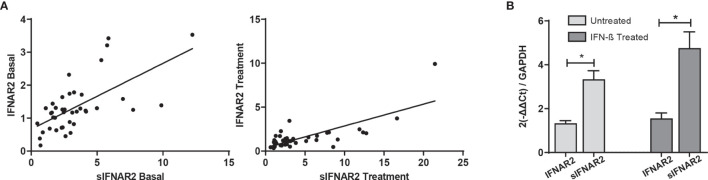
IFNAR2 splice variants under IFN-β treatment. **(A)** Correlation analysis between the transmembrane (IFNAR2) and soluble (sIFNAR2) transcripts before (r=0.636 p< 0.001) and after 6 months of IFN-β therapy onset (r=0.749 p<0.001). **(B)** Comparison of the relative expression of IFNAR2 versus sIFNAR2 before and after 6 months of IFN-β therapy onset, using GAPDH as a raeference gene (N=41). Significant p-values are shown with asterisk.

### IFNAR2 Splice Variants According to the IFN-β Response

**Figure 5:**
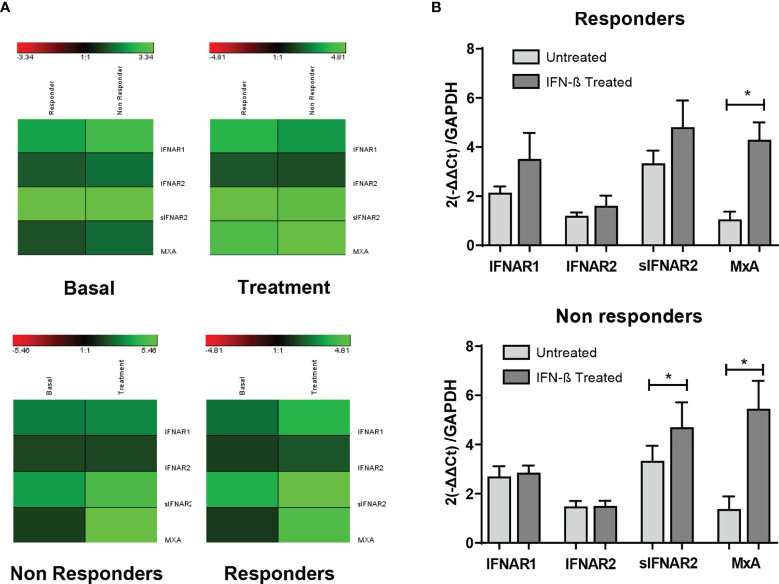
Longitudinal assessment of IFNAR1, IFNAR2, sIFNAR2 and MxA gene expression in responder and non-responder patients. **(A)** Heat map representing the gene expression data of IFNAR1, IFNAR2, sIFNAR2, and MxA between responder and non-responder patients, at baseline and 6 months after the onset of IFN-β therapy. Unchanged proteins are displayed in black, up-regulated proteins are displayed in green while the down-regulated proteins are depicted in red. **(B)** Bar chart representing the gene expression for responders (N=22) and non-responders (N=19) using GAPDH as raeference gene. Significant p-values are shown with asterisk.

The gene expression data of IFNAR1, IFNAR2, sIFNAR2 and MxA have been represented using a heat map (**Figure 5**). Comparison between responders and non-responders, showed no differences in the expression of any of the genes at baseline and after 6 months of therapy (A). The comparison between baseline and that after 6-month of therapy in both groups of patients (B) showed an increase more accentuated in responders after treatment, but there were not statistical differences in IFNAR1 and IFNAR2 gene expression. However, MxA expression increased significantly after treatment independently of the therapeutic response (p<0.001 and p=0.006, in responders and non-responders, respectively) and sIFNAR2 also increased in both groups of patients although significance was reached only in non-responders (p=0.020).

**Figure 5**

### Induction of Proteases by IFN-β

In order to evaluate the ability of IFN-β to induce the expression of proteases that have been described to cleave the transmembrane IFNAR2 subunit, the expression of ADAM17 and PSEN 1 and 2 have been measured at baseline and after 6 months of IFN-β therapy in patients of cohort 1. There was no increase in the expression of ADAM17 and PSEN 2 after treatment, however there was a significant increase in the expression of PSEN 1 (p=0.041).

**Figure 6**

**Figure 6:**
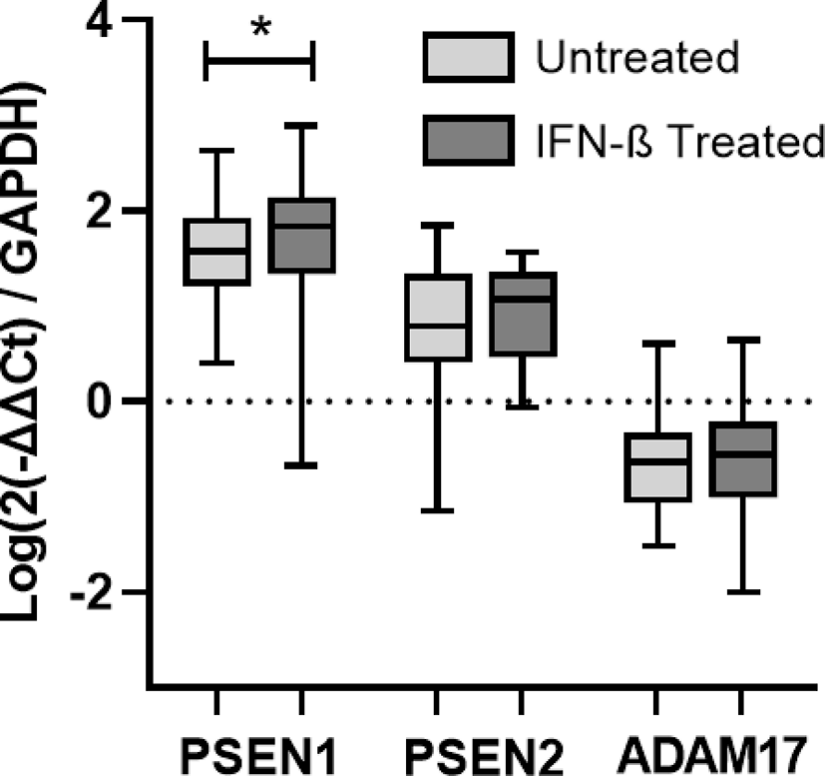
Longitudinal assessment of PSEN1, PSEN2, and ADAM17 gene expression. RNA relative expression of PSEN1, PSEN2, and ADAM17 in PBMC from MS patients before and after 6 months of IFN-β treatment onset (N=41), assessed by real time-PCR using GAPDH as raeference gene. Significant p-values are shown with an asterisk. Logarithms are used to normalize and scale data for representation.

### sIFNAR2 Gene Expression Increases After Short-Term IFN-β Induction *In Vitro*

PBMC isolated from seven untreated MS patients were cultured to demonstrate the short-term induction of sIFNAR2 by IFN-β. MxA was also measured as positive control since it is well known to be induced by IFN-β (31). As expected, its expression was significantly increased after 8 hours of IFN-β induction (p=0.043). There was no change in the expression of sIFNAR2 after 8 hours of IFN-β induction, however all patients increased their sIFNAR2 expression after 24 h of induction (p=0.043), with the exception of one of them.

**Figure 7**

**Figure 7:**
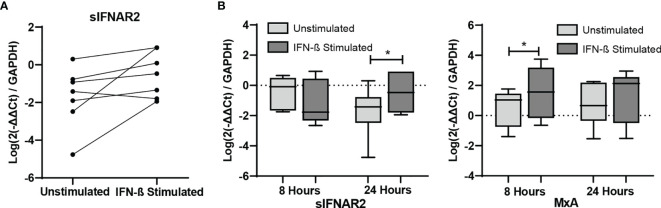
*In vitro* study of IFNAR1, IFNAR2, sIFNAR2 and MxA gene expression. Representation of RNA relative expression of IFNAR1, IFNAR2, sIFNAR2 and MxA in PBMC from untreated MS patients cultured with or without IFN-β stimulus (N=7) during 8 and 24 hours. **(A)** sIFNAR2 expression in PBMC from each patient after 24 hours of culture in the presence or in the absence of IFN-β. **(B)** Boxplots comparing sIFNAR2 and MxA expression in unstimulated PBMC and IFN-β-stimulated PBMC during 8 and 24 hours of culture. Significant p values are marked with an asterisk. Logarithms are used to normalize and scale data for representation.

## Discussion

The treatment landscape has changed in MS over the past two decades and, currently, many drugs are approved for RRMS (32). Most of the recent drugs target specific molecules as a result of a better knowledge of the pathophysiology of the disease and have a rational drug design. However, IFN-β preparations have no clearly defined mechanisms of action despite being the first approved drug by the US Food and Drug Administration (FDA) for the treatment of MS in 1993, and continue being a first line treatment for MS. IFN-β plays an important role in the regulation of the immune system and has a wide range of immunomodulatory effects, but how these effects translate into the beneficial therapeutic effect in MS is still not understood. However, IFN-β can also have adverse effects (injection site reactions, nausea, headaches, fever, leukopenia, and other more serious although rare) (33); therefore, biomarkers to predict treatment response could avoid these undesirable side effects in patients that may not benefit of the treatment. Alternative splicing is a mechanism for regulating gene expression that allows a single gene to code for multiple protein isoforms. It occurs in up to 94% of human genes and the isoforms generated may have related, distinct or even opposing functions (9). Many relevant genes of the immune system have been shown to undergo alternative splicing, although the knowledge of how this mechanism may regulate the immune system are still limited (7). Nevertheless, an altered RNA of genes mediating immune signaling pathways has been repeatedly implicated in MS pathogenesis (34).

sIFNAR2 is able to bind circulating IFN-β and modulate its activity, as it may have agonist or antagonist properties (20). However, its role in the immune system is mostly unknown, despite that the human form was first cloned in 1995 (15). Therefore, we cloned a recombinant form analogous to human soluble IFNAR2 and recently demonstrated that it exerts antiviral, immunomodulatory and antiproliferative activities without IFN-β mediation (35). Regarding the native form, we developed an ELISA to detect sIFNAR2 in serum that was analytically validated (26). This ELISA uses a double antigen recognition and detects sIFNAR2 generated by both mechanisms, alternative splicing and proteolysis (26). Moreover, we observed that sIFNAR2 increased significantly in IFN-β-treated patients during the first year of therapy, in contrast to GA and natalizumab-treated patients (23). Herein, we assessed in depth the ability of IFN-β treatment to induce the production of sIFNAR2 and its potential relationship with the heterogeneity of the clinical response in MS patients treated with IFN-β.

First, we analyzed the sIFNAR2 serum levels in a follow up study of two independent cohorts of MS patients and observed an increase of sIFNAR2 levels after 6 months of IFN-β administration in both cohorts. As aforementioned, IFN-β is able to bind either their membrane-bound receptors or their soluble counterparts (20), consequently, the action of the IFN-β could ultimately depend on the relative abundance of each isoform. Although there are soluble receptors that enhance the effect of their ligand, the majority of the known soluble cytokine receptors has antagonistic functions (4), which could explain that non-responder patients increased significantly sIFNAR2 levels at 6 months, in contrast to responder patients, who showed a non-significant increase. Interestingly, a study published in 1999 suggested that high serum levels of soluble sIFNAR2 suppressed the effectiveness of IFN therapy in patients with chronic hepatitis C (36). Both studies suggest an antagonist effect of sIFNAR2 at high levels that could have important implications if the IFN-β activities are neutralized, as previously described *in vitro* by *Mckenna et al.* (21). However, it should be considered that an opposite effect could be reached at low concentrations (21), being proposed as an important agonist of endogenous IFN actions in pathophysiological processes like septic shock (22). Beyond these studies, there are no recent research focused on the sIFNAR2/IFN-β relationship, which would be necessary for the optimization of the IFN-β treatment given the agonist and antagonist properties of sIFNAR2.

Regarding the gene expression follow-up study, the functional subunits of the receptor, IFNAR1 and IFNAR2, showed a slight increase after IFN-β therapy, while MxA increased significantly its expression, as expected. sIFNAR2 gene expression behaved similar to MxA, being significantly induced after treatment with IFN-β. This up-regulation in the levels of sIFNAR2 over the first year of therapy was previously described by Gilli et al. in MS patients that were negative for neutralizing antibodies. According to the IFN-β clinical response, responder and non-responder patients increased sIFNAR2 after treatment but only non-responders reached statistical significance, in line to which was observed at protein levels. It has been described that non-responders had an activated type I IFN system in peripheral blood cells that was refractory to exogenous IFN-β (37). The elevated levels of circulating sIFNAR2 found in our study, acting as an antagonist to IFN-β, could be a plausible explanation of that absence of response observed in non-responders, but this has to be further studied.

As the transmembrane IFNAR2 and sIFNAR2 are products of an alternative splicing, we further analyzed the relationship between both spliced variants with the treatment. The relative expression of the transmembrane protein and its soluble counterpart showed a strong positive correlation, which was not modified by the IFN-β treatment. It should be highlighted that the expression of the sIFNAR2 was 2.3-fold higher compared to the transmembrane isoform before and after IFN-β therapy. The greater abundance of the mRNA transcript for the soluble isoform than for the transmembrane one was previously described in mouse tissues, where both transcripts were expressed ubiquitously and the ratio soluble:transmembrane IFNAR varied from 10:1 in the liver and other organs, to 1:1 in tissues involved in hematopoiesis, suggesting that both receptor isoforms should be regulated independently (20). However, a comprehensive study of human sIFNAR2 mRNA expression in normal tissue is still lacking, and hence there is limited information on normal relative expression levels.

As aforementioned, in addition to the alternative splicing, the soluble receptors of cytokines can also be generated by proteolytic cleavage. Cell membrane proteins undergo this cleavage at the cell surface, resulting in the release of a significant portion of their extracellular domain (11). ADAM17, proteolytically cleaves many substrates including the IFNAR2 receptor (17, 18). This led us to think that the serum levels of sIFNAR2 detected in MS patients after one year of IFN-β exposure could be the result of both alternative splicing but also importantly of proteolytic cleavage (or shedding). However, we did not find any induction of ADAM17 after IFN-β administration which suggests that serum sIFNAR2 was most probably the product of alternative splicing of the IFNAR2 gene rather than a proteolytically cleaved product of IFNAR2, in concordance with data observed in mouse (38). Regarding the presenilins, it has been described that they release the intracellular domain of IFNAR2 (18), although it is not clear whether this cleavage leads also to the release of the extracellular domain. We have found a significant increase in the expression of PSEN 1 in MS patients after IFN-β treatment, but its implication on the release of the intracellular domain of IFNAR2 is unknown and should be further studied.

We finally determined whether *in vitro* short-term IFN-β induction modified the sIFNAR2 gene expression in PBMC from MS patients. Increased expression of MxA after the short-term IFN-β induction was observed and considered as the positive control of the experiment. No changes were observed after 8 hours of IFN-β administration in the expression of sIFNAR2, but a significant increase was observed at 24 hours. This upregulation of sIFNAR2 mRNA was previously described in the murine cell line L929 after induction with type I and II IFN (38). Herein, we describe for the first time the up-regulation of sIFNAR2 mRNA in human PBMC, which could have important implications for IFN-β treatment optimization, due to the fact that it can act as an agonist or antagonist depending on its circulating levels.

Beyond MS, IFN-β is a pleiotropic cytokine, collectively having roles in both innate and adaptive immune responses. Thereby the function of IFNβ-sIFNAR2 mediated regulatory mechanisms could also exerts an important role in other diseases characterized by a dysregulation of the interferon pathway, named interferonopathies (39), although the complete functionality of sIFNAR2 in type I IFN signal transduction requires further elucidation.

A limitation of the study is that it is not possible to know if the absence of clinical response to IFN-β is the cause or the consequence of the up-regulation of sIFNAR2, so further studies including patients during the relapse and in remission are warranted. Also, it would necessary to carry out a replication study in a large cohort of responders and non-responders to confirm its use as biomarker of response. If confirmed, sIFNAR2 would be an easy ideal biomarker to implement in clinical practice because its determination is made by ELISA, which is a widely used technique in clinical laboratories, it requires a non-invasive method for the sample extraction and it is cost-effective.

In conclusion, our study demonstrates the induction of sIFNAR2 (protein and mRNA) by IFN-β administration, probably by inducing alternative splicing, which at higher levels it might be related to the reduction or absence of therapeutic response to IFN-β treatment. IFN-β activity should be tightly regulated and sIFNAR2 levels could open a new dimension for optimizing this treatment and give another opportunity for improved therapeutic interventions.

## Data Availability Statement

The raw data supporting the conclusions of this article will be made available by the authors, without undue reservation.

## Ethics Statement

The studies involving human participants were reviewed and approved by Comité de Ética de la Investigación Málaga Nordeste. The patients/participants provided their written informed consent to participate in this study.

## Author Contributions

Conceptualization, BO-M and OF. Funding acquisition, BO-M. Methodology and data acquisition, PA-G, IH-G, JR-B, and I-BM. Patients’ recruitment, NC-P, PU, VR, AA, RA, and LR-T. Data analysis, BO-M, PA-G, and IH-G. Writing – original draft, BO-M, IH-G, and PA-G. Writing –review and editing, OF, LL, RA-L, and EQ. All authors contributed to the article and approved the submitted version.

## Funding

This research was funded by grants from the Instituto de Salud Carlos III and co-funded by European Regional Development Fund (ERDF), Technological Development Project in health DTS/1800045 to BO-M. BO-M holds a contract from Red Andaluza de Investigacion Clínica y Traslacional en Neurología (Neuro-reca) (RIC-0111-2019). PA-G is supported by Promoción de Empleo Joven e Implantación de la Garantía Juvenil 2018 (PEJ2018-002719-A). JR-B is supported by grants from Red Temática de Investigación Cooperativa, Red Española de Esclerosis Multiple REEM (RD16/0015/0010). LL holds a Nicolás Monardes research contract (RC-002-2019) from the Andalusian Ministry of Health and Family. IB-M holds a pFIS contract (FI19/00139) from the Spanish Science and Innovation Ministry.

## Conflict of Interest

The authors declare that the research was conducted in the absence of any commercial or financial relationships that could be construed as a potential conflict of interest.

## Publisher’s Note

All claims expressed in this article are solely those of the authors and do not necessarily represent those of their affiliated organizations, or those of the publisher, the editors and the reviewers. Any product that may be evaluated in this article, or claim that may be made by its manufacturer, is not guaranteed or endorsed by the publisher.
