# Supplementary material for: Soluble Receptor Isoform of IFN-Beta (sIFNAR2) in Multiple Sclerosis Patients and Their Association With the Clinical Response to IFN-Beta Treatment
Source: Front Immunol. 2021 Dec 16;12:778204. doi: 10.3389/fimmu.2021.778204 (PMC8716373; doi:10.3389/fimmu.2021.778204)

Supplementary Figure 1. sIFNAR2 primer sequence design.

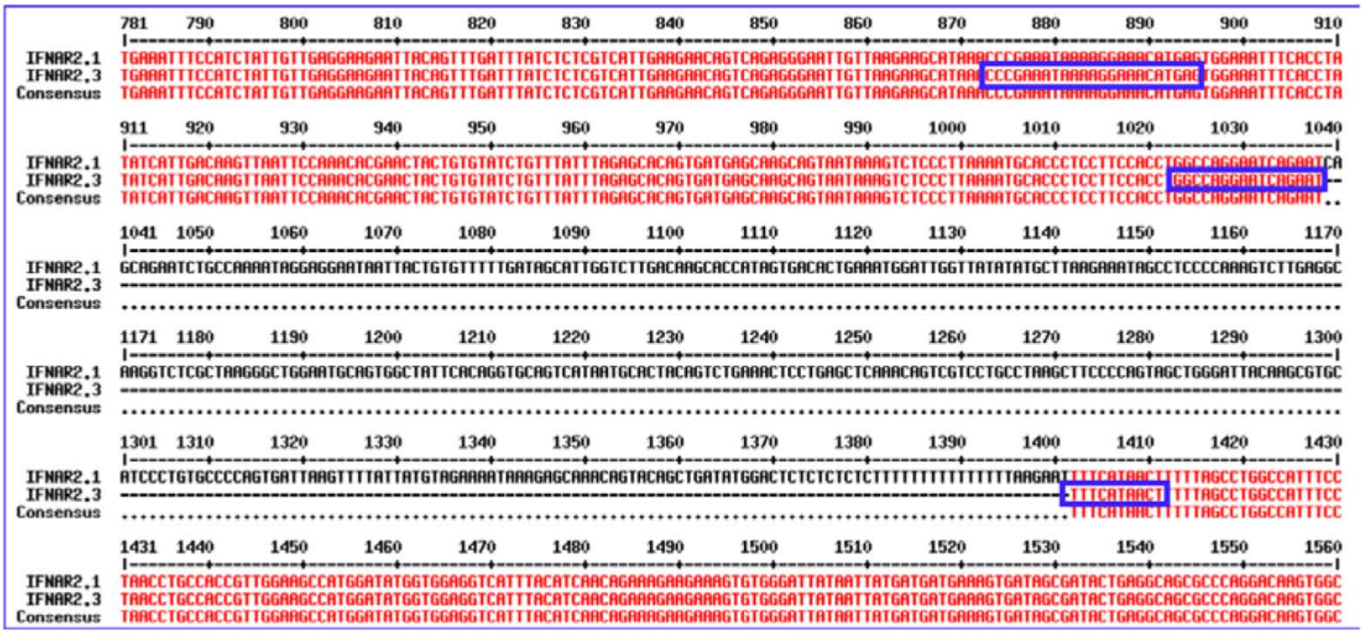

Supplementary Table 1. Primer information and/or sequence.

Gene name	DNA sequence / Catalog number
IFNAR1	Cat. no: PPH00869F-200 QIAGEN
IFNAR2	Cat. no: PPH00870B-200 QIAGEN
MxA	Cat. no: PPH01325A-200 QIAGEN
PSEN1	Cat. no: QT00001862 QIAGEN
PSEN2	Cat. No: QT00196854 QIAGEN
GAPDH	Cat. no: PPH00150F-200 QIAGEN
sIFNAR2	Sense: 5' CCCGAAATAAAAGGAAACATGA 3'<br>Antisense: 5' GGCCAGGAATCAGAATTTTCATAACT 3'
ADAM17	Sense: 5' CCCCGATGTGAGCAGTTT 3'<br>Antisense: 5' AATCAAGCTTCTCGAGTCTCT 3'

Supplementary Figure 2. ΔsIFNAR2 for each patient after 6 or 12 months of IFN-ß treatment onset in (A) Cohort 1 and (B) Cohort 2.

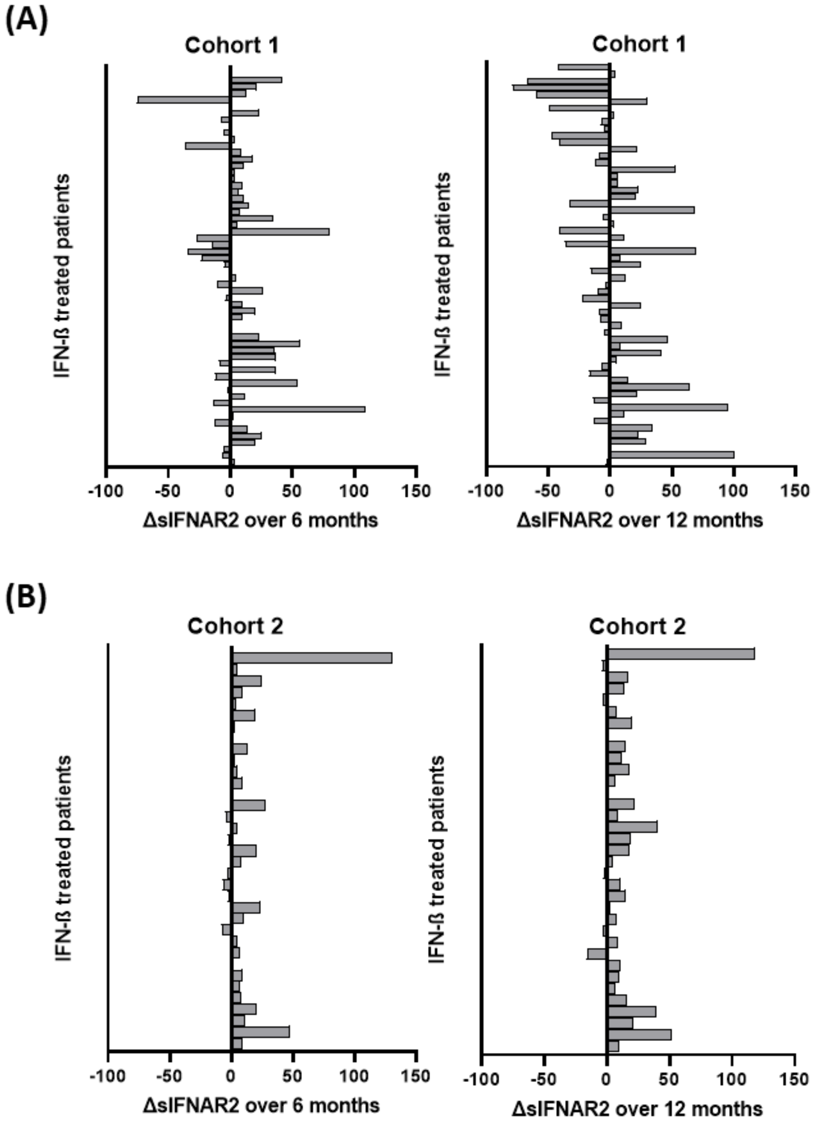

Supplement: Supplementary file 1 [file DataSheet_1.pdf]
